# Supplementary material for: Resolving the full spectrum of human genome variation using Linked-Reads
Source: Genome Res. 2019 Apr;29(4):635–45. doi: 10.1101/gr.234443.118 (PMC6442396; doi:10.1101/gr.234443.118)
Supplement: Supplemental Material [file supp_gr.234443.118_Supplemental_longranger-2.2.zip › longranger-2.2.tar.bz2!/lr2.2-public/lib/stan/cmdstan/stan/doc/api/html/math_2matrix_2_l_d_l_t__factor_8hpp_source.html]

Stan: src/stan/math/matrix/LDLT\_factor.hpp Source File


|  |  |
| --- | --- |
|  | Stan  2.5.0  probability, sampling & optimization |


- Main Page
- Related Pages
- Namespaces
- Classes
- Files

- File List
- File Members

All Classes Namespaces Files Functions Variables Typedefs Enumerations Enumerator Friends Macros Pages

- src
- stan
- math
- matrix

LDLT\_factor.hpp

Go to the documentation of this file.

1 #ifndef STAN\_\_MATH\_\_MATRIX\_\_LDLT\_FACTOR\_HPP

2 #define STAN\_\_MATH\_\_MATRIX\_\_LDLT\_FACTOR\_HPP

3

4 #include <stan/math/matrix/Eigen.hpp>

5 #include <boost/shared\_ptr.hpp>

6 #include <stan/math/error\_handling/matrix/check\_square.hpp>

7

8 namespace stan {

9  namespace math {

10

11  // This class is conceptually similar to the corresponding Eigen class

12  // Any spd matrix A can be decomposed as LDL' where L is unit lower-triangular

13  // and D is diagonal with positive diagonal elements

14

15  template<typename T, int R, int C>

16  class LDLT\_factor;

17

55  template<int R, int C, typename T>

56  class LDLT\_factor<T,R,C> {

57  public:

58  LDLT\_factor()

59  : N\_(0), \_ldltP(new Eigen::LDLT< Eigen::Matrix<T,R,C> >()) {}

60

61  LDLT\_factor(const Eigen::Matrix<T,R,C> &A)

62  : N\_(0), \_ldltP(new Eigen::LDLT< Eigen::Matrix<T,R,C> >())

63  {

64  compute(A);

65  }

66

67  inline void compute(const Eigen::Matrix<T,R,C> &A) {

68  stan::math::check\_square("LDLT\_factor(%1%)",A,"A",(T\*)0);

69  N\_ = A.rows();

70  \_ldltP->compute(A);

71  }

72

73  inline bool success() const {

74  bool ret;

75  ret = \_ldltP->info() == Eigen::Success;

76  ret = ret && \_ldltP->isPositive();

77  ret = ret && (\_ldltP->vectorD().array() > 0).all();

78  return ret;

79  }

80

81  inline T log\_abs\_det() const {

82  return \_ldltP->vectorD().array().log().sum();

83  }

84

85  inline void inverse(Eigen::Matrix<T,R,C> &invA) const {

86  invA.setIdentity(N\_);

87  \_ldltP->solveInPlace(invA);

88  }

89

90  template<typename Rhs>

91  inline const Eigen::internal::solve\_retval<Eigen::LDLT< Eigen::Matrix<T,R,C> >, Rhs>

92  solve(const Eigen::MatrixBase<Rhs>& b) const {

93  return \_ldltP->solve(b);

94  }

95

96  inline Eigen::Matrix<T,R,C> solveRight(const Eigen::Matrix<T,R,C> &B) const {

97  return \_ldltP->solve(B.transpose()).transpose();

98  }

99

100  inline Eigen::Matrix<T,Eigen::Dynamic,1> vectorD() const {

101  return \_ldltP->vectorD();

102  }

103

104  inline Eigen::LDLT<Eigen::Matrix<T,R,C> > matrixLDLT() const {

105  return \_ldltP->matrixLDLT();

106  }

107

108  inline size\_t rows() const { return N\_; }

109  inline size\_t cols() const { return N\_; }

110

111  typedef size\_t size\_type;

112  typedef double value\_type;

113

114  size\_t N\_;

115  boost::shared\_ptr< Eigen::LDLT< Eigen::Matrix<T,R,C> > > \_ldltP;

116  };

117  }

118 }

119 #endif

stan::math::LDLT\_factor< T, R, C >::inverse

void inverse(Eigen::Matrix< T, R, C > &invA) const

**Definition:** LDLT\_factor.hpp:85

stan::math::LDLT\_factor< T, R, C >::vectorD

Eigen::Matrix< T, Eigen::Dynamic, 1 > vectorD() const

**Definition:** LDLT\_factor.hpp:100

N\_

int N\_

**Definition:** mdivide\_left.hpp:20

stan::math::LDLT\_factor< T, R, C >::\_ldltP

boost::shared\_ptr< Eigen::LDLT< Eigen::Matrix< T, R, C > > > \_ldltP

**Definition:** LDLT\_factor.hpp:115

stan::math::LDLT\_factor< T, R, C >::value\_type

double value\_type

**Definition:** LDLT\_factor.hpp:112

stan::math::LDLT\_factor< T, R, C >::LDLT\_factor

LDLT\_factor()

**Definition:** LDLT\_factor.hpp:58

stan::math::LDLT\_factor< T, R, C >::LDLT\_factor

LDLT\_factor(const Eigen::Matrix< T, R, C > &A)

**Definition:** LDLT\_factor.hpp:61

stan::math::transpose

Eigen::Matrix< T, C, R > transpose(const Eigen::Matrix< T, R, C > &m)

**Definition:** transpose.hpp:12

stan::math::LDLT\_factor

**Definition:** LDLT\_factor.hpp:16

stan::math::LDLT\_factor< T, R, C >::success

bool success() const

**Definition:** LDLT\_factor.hpp:73

stan::math::LDLT\_factor< T, R, C >::solve

const Eigen::internal::solve\_retval< Eigen::LDLT< Eigen::Matrix< T, R, C > >, Rhs > solve(const Eigen::MatrixBase< Rhs > &b) const

**Definition:** LDLT\_factor.hpp:92

stan::math::LDLT\_factor< T, R, C >::log\_abs\_det

T log\_abs\_det() const

**Definition:** LDLT\_factor.hpp:81

stan::math::LDLT\_factor< T, R, C >::matrixLDLT

Eigen::LDLT< Eigen::Matrix< T, R, C > > matrixLDLT() const

**Definition:** LDLT\_factor.hpp:104

stan::math::LDLT\_factor< T, R, C >::N\_

size\_t N\_

**Definition:** LDLT\_factor.hpp:114

Eigen.hpp

\_ldltP

boost::shared\_ptr< Eigen::LDLT< Eigen::Matrix< double, R1, C1 > > > \_ldltP

This share\_ptr is used to prevent copying the LDLT factorizations for mdivide\_left\_ldlt(ldltA,b) when ldltA is a LDLT\_factor<double>.

**Definition:** mdivide\_left\_ldlt.hpp:23

stan::math::LDLT\_factor< T, R, C >::size\_type

size\_t size\_type

**Definition:** LDLT\_factor.hpp:111

stan::math::LDLT\_factor< T, R, C >::rows

size\_t rows() const

**Definition:** LDLT\_factor.hpp:108

stan::math::LDLT\_factor< T, R, C >::compute

void compute(const Eigen::Matrix< T, R, C > &A)

**Definition:** LDLT\_factor.hpp:67

stan::math::LDLT\_factor< T, R, C >::solveRight

Eigen::Matrix< T, R, C > solveRight(const Eigen::Matrix< T, R, C > &B) const

**Definition:** LDLT\_factor.hpp:96

stan::math::LDLT\_factor< T, R, C >::cols

size\_t cols() const

**Definition:** LDLT\_factor.hpp:109

stan::math::check\_square

bool check\_square(const char \*function, const Eigen::Matrix< T\_y, Eigen::Dynamic, Eigen::Dynamic > &y, const char \*name, T\_result \*result)

Return true if the specified matrix is square.

**Definition:** check\_square.hpp:24

check\_square.hpp


---

[ Stan Home Page ]

*© 2011–2014,
Stan Development Team.*
